# Bottom-Up Approaches to Synthetic Cooperation in Microbial Communities

**DOI:** 10.3390/life9010022

**Published:** 2019-02-26

**Authors:** Daniel Rodríguez Amor, Martina Dal Bello

**Affiliations:** Physics of Living Systems, Department of Physics, Massachusetts Institute of Technology, Cambridge, MA 02139, USA; dalbello@mit.edu

**Keywords:** synthetic microbial communities, mutualism, cheaters, host-microbiome interactions, synthetic ecology

## Abstract

Microbial cooperation pervades ecological scales, from single-species populations to host-associated microbiomes. Understanding the mechanisms promoting the stability of cooperation against potential threats by cheaters is a major question that only recently has been approached experimentally. Synthetic biology has helped to uncover some of these basic mechanisms, which were to some extent anticipated by theoretical predictions. Moreover, synthetic cooperation is a promising lead towards the engineering of novel functions and enhanced productivity of microbial communities. Here, we review recent progress on engineered cooperation in microbial ecosystems. We focus on bottom-up approaches that help to better understand cooperation at the population level, progressively addressing the challenges of tackling higher degrees of complexity: spatial structure, multispecies communities, and host-associated microbiomes. We envisage cooperation as a key ingredient in engineering complex microbial ecosystems.

## 1. Introduction

Cooperation emerges at multiple scales of complexity in microbial ecosystems. Clonal populations are among the simplest microbial ecosystems that can be studied, and yet, they provide a convenient laboratory arena to analyze several cooperative behaviors in microbes [1,2]. These include extracellular digestion of resources [3,4], protection against antibiotics [5], and even the formation of fruiting bodies, a much rarer event that enhances the fitness of a small fraction of the population at the expense of the majority [6]. Inspection of natural communities reveals widespread cooperative interactions occurring not only within cells sharing a genotype [7], but also between different strains or species; see Table 1. Such heterotypic interactions are commonly known as mutualisms, and they can give rise to a variety of behaviors in microbial consortia, e.g., cross-feeding [8], cross-protection [9], and division of labor [10]. These behaviors can be influenced by specific lifestyles that microbial communities adopt, such as the formation of spatially-structured biofilms [11]. In a way, microbial cooperative skills can even transcend the small size of unicellular organisms as, for example, in microbiomes, where microbes engage in symbiotic relationships with their hosts.

Despite the progresses made in the past decades in disentangling microbe–microbe and host–microbe interactions, we still have limited understanding of microbial cooperation in natural communities [12]. What mechanisms promote cooperative interactions? How does cooperation shape the dynamics of complex microbial communities, or more generally, how can cooperators endure exploitation by cheaters? Since *The Origin of Species* was published, this question has puzzled evolutionary scientists [13,14,15,16,17,18], Charles Darwin included. Assuming a simple scenario in which cooperators pay a fitness cost in order to help their neighbors altruistically, cheaters could easily beat cooperators by exploiting any available public good while avoiding its costs. Hence, for cooperation to be an evolutionarily-stable strategy, additional mechanisms promoting cooperation have to be at play [18,19].

The recent blooming of synthetic biology [20] has provided a convenient platform to interrogate cooperation in microbial systems. Editing wild strain genomes has allowed manipulating microbial strategies within a population in order to understand how microbes face social dilemmas [1,21,22,23,24,25]. Moreover, engineering mutualisms between multiple genotypes recently provided insights into heterotypic partnerships such as cross-feeding interactions [8], collective resistance to antibiotics [9], and spatial self-organization [26]. Engineered symbiosis is progressively opening new avenues to explore interactions that benefit both microbial consortia and their associated hosts. Beyond improving our understanding of microbial interactions, a major goal in synthetic biology is to engineer complex microbial ecosystems for industrial [27,28], bioremediation [29], or therapeutic purposes [30,31]. To this aim, a better understanding of how cooperative feedbacks could be used to enhance the productivity and stability of different engineered consortia is needed.

**Figure 1:**
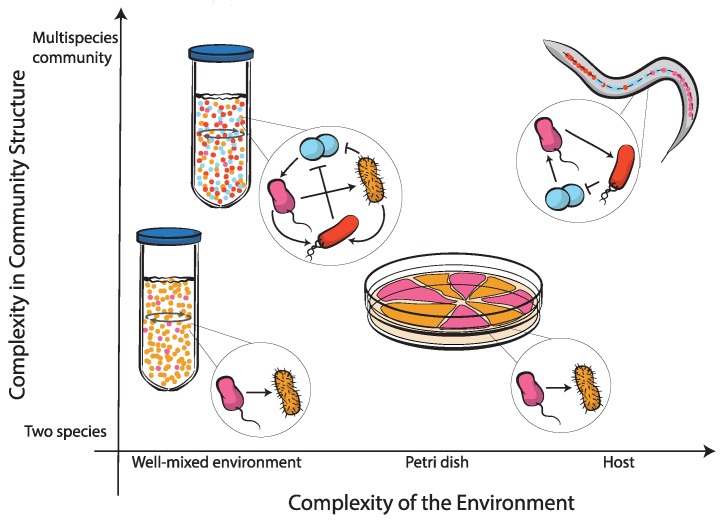
The complexity of microbial ecosystems classified according to two different components: the structure of the community and the structure of the environment. As the number of species in the community increases, community structure (and hence, the network of interactions) becomes more complex. As the complexity of the environment increases, the ecosystem becomes more heterogeneous, very often unfolding new outcomes for the community. A well-mixed culture with two strains (bottom-left) provides one of the simplest ways to study microbial interactions, yet the outcome of interactions can change if spatial structure is at play (agar surface at the bottom-right). The complexity of the interaction network can increase with the number of community members (top-left), and again, complex environments such a spatially-structured animal gut can interfere with both microbial interactions and community composition (top-right).

Here, we review recent advances on synthetic cooperation in microbial ecosystems. We focus on cooperative and parasitic interactions (see Table 1) in synthetic microbial ecosystems from an ecological perspective, rather than focusing on the specific genetic circuits to engineer these systems, which were reviewed, e.g., by McCarty et al. [32] and by Brophy et al. [33]. In the following, we start by discussing low complexity systems in simple laboratory environments, progressively moving on to more complex microbial ecosystems (see Figure 1). Each of the following sections covers a specific scale of complexity: well-mixed populations, spatially-structured environments, multispecies communities, and host-associated microbial communities. While reviewing several key drivers of microbial cooperation at these different scales, we highlight the potential impact of cheaters that exploit collective benefits. We discuss several mechanisms that promote cooperation against cheaters in microbial ecosystems, as well as how transitions between cooperators and cheaters could be used in microbial community engineering.

## 2. Engineering Microbial Cooperation, Mutualism and Cheating in Well-Mixed Environments

Synthetic biology has chiefly helped to reveal different mechanisms that enhance cooperation in the microbial world. We devote this section to reviewing synthetic cooperation in the simplest microbial ecosystems: well-mixed populations with minimal, or low phenotypic diversity.

**Figure 2:**
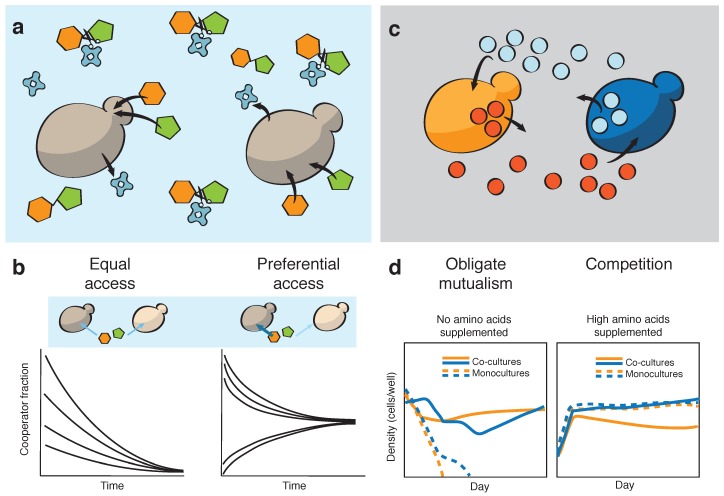
Cooperation and mutualism in a synthetic yeast model. (**a**) Yeast cells cooperate during extracellular digestion of sucrose via secretion of invertase. (**b**) Preferential access to the public goods leads to coexistence between cooperators (dark gray cells) and cheaters (light gray) in synthetic yeast populations [21]. (**c**) Auxotrophic yeast strains can engage in mutualism by cross-feeding essential amino acids through the environment (**d**) Environmental conditions can drive microbial interactions. In a medium lacking amino acids, two auxotrophic strains need to cooperate with each other to survive (obligate mutualism). Instead, competition for available resources becomes the driving interaction when the medium is supplemented with enough amino acids [37].

The budding yeast *Saccharomyces cerevisiae* is one of the most famous model systems to study microbial cooperation. When growing on sucrose as the only carbon source, yeast cells cannot directly uptake the complex sugar, but need to break it down into fructose and glucose, two simple sugars that can be transported through the cell membrane. Growth becomes cooperative, as yeast cells release the enzyme invertase that digests the sucrose in the extracellular medium, as shown in Figure 2a. Given that cooperative cells endure a fitness cost to produce the invertase, what prevents a cheater strain from taking over a population of cooperators? Gore et al. [21] showed that preferential access to the public good allows these microbial cooperators to endure exploitation by cheaters. For this purpose, the authors engineered a cheater strain that was unable to produce invertase due to a SUC2 gene knockout. The cooperative strain had instead an intact SUC2 gene, but an engineered auxotrophy for histidine, which allowed tuning its relative fitness in different experiments in which the two strains were cocultured. These experiments showed that, as cooperators release the invertase, sucrose digestion occurs at a slightly higher rate in the surroundings of their cell membrane, which gives cooperators a small advantage at capturing the simple sugars. This provides a fitness advantage to cooperators when they are rare, because simple sugars become scarce as few invertase is released to the medium. However, when the fraction of cooperators in the population is high—leading to high invertase concentration in the medium and higher availability of simple sugars—avoiding the cost of invertase production pays off and gives cheaters an advantage. As a result, these synthetic yeast populations exhibit coexistence between cooperators and cheaters; see Figure 2b. While neither cheaters nor cooperators can take over the population in this case, the stable fraction can respond to different features such as eco-evolutionary feedbacks [35] or changes in environmental conditions (e.g., the fraction of cooperators increases in harsher environments [36]).

Microbes often engage in cooperative feedbacks with genetically-different cells, establishing mutualistic interactions [38,39]. In particular, cross-feeding mutualisms in which cells exchange essential metabolites are very common in microbial communities [2,40,41]. Such exchanges typically involve leakage and uptake of mutualistic metabolites: each mutualist secretes chemicals that benefit its partner, so that the counterpart can uptake them from the extracellular medium. A pioneer work by Shou et al. [8] showed that two engineered auxotrophic strains could sustain growth through such a mutualistic interaction (Figure 2c). For these synthetic yeast strains, however, engineered overproduction of the amino acid needed by the partner strain was necessary for an effective mutualistic growth. Recent studies have revealed additional examples of engineered mutualisms, not only based on cross-feeding of different metabolites [39,42,43,44], but also through other mechanisms such as antibiotic cross-protection [9]. Even in the absence of external threats by (third-party) competitors or environmental fluctuations, mutualists can exhibit complex population dynamics including oscillations [9] and rapid adaptation to first encounters [45].

Metabolites that drive cooperation are very often traded through the microbial environment [22], potentially compromising the stability of cooperative populations against environmental changes. This question was explored in Hoek et al. [37], where the authors drove two synthetic yeast strains through a wide range of social interactions as the environment was modified. Each of these strains had been engineered in order to overproduce either leucine or tryptophan, while lacking the metabolic pathway for the other amino acid. In response to the amino acid concentrations that were supplemented to the medium, the strains gradually transitioned from a cross-feeding mutualism to parasitism and to competition (Figure 2d). These results highlight the plasticity of microbial interactions, as well as the potential to engineer microbial interactions through environmental tuning.

From an evolutionary perspective, the conditions in which the emergence of microbial mutualism should be expected are still under debate. On the one hand, minimizing metabolic costs via division of labor could favor the appearance of mutualisms [43]. For example, multi-genome in silico analysis suggests that coevolution could frequently lead to cooperative interdependencies resulting from gene loss [46]. On the other hand, an evolutionary race towards minimum metabolic costs could instead promote hierarchical ecosystems in which just one producer strain cross-feeds metabolites with different kinds of cheaters. The latter mechanism is known as the Black Queen hypothesis [47,48]. Predicting whether a specific evolutionary scenario would unfold in a given microbial ecosystem is nevertheless extremely challenging. As evolving metabolisms are likely to impact their environment differently, the resulting changes in the fitness landscape can significantly affect the fate of later descendants, making long evolutionary trajectories especially difficult to predict [49]. Developing a better understanding of dynamic fitness landscapes will certainly help to unveil how microbial interactions emerge and develop under eco-evolutionary feedbacks.

## 3. The Role of Spatial Structure

Although cheaters often constitute an important threat to microbial mutualists in the well-mixed arena [50,51], mutualists can still thrive over cheaters in more complex scenarios, e.g., those displaying a spatial structure. Microbes self-organize in space and, as a result, microbial populations can exhibit enhanced heterogeneity in, for example, interactions with neighboring cells, the availability of resources, and exposure to waste and toxins. Within spatially-structured biofilms [11], these self-organization capabilities allow microbial cells to achieve emergent collective features such as strong antibiotic resistance, waste degradation, or nutrient recycling.

**Figure 3:**
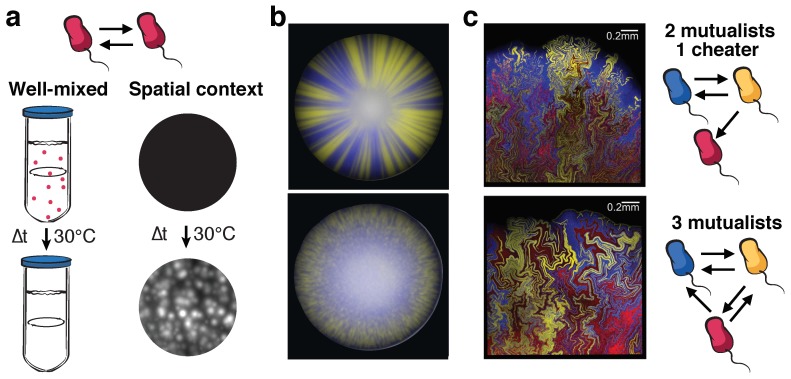
Spatial structure of cooperators and mutualists. (**a**) Clustering of cooperators allows survival in challenging environments. A small-sized inoculum of *B. subtilis* cells reaches extinction in well-mixed conditions as the low number of initial cooperators is unable to perform an effective extracellular digestion of starch to support growth. When the same amount of cells is inoculated in the spatial context of an agar surface, clusters of cooperators arise and grow, since extracellular digestion becomes locally more efficient around these groups of cooperators (adapted from Ratzke and Gore [52]). (**b**) Competition leads to segregation of phenotypes (top), while mutualism promotes phenotypic intermixing (bottom) in growing yeast colonies (adapted from Muller et al. [44]). (**c**) Cheaters are progressively left behind as bacterial mutualists expand into available space (top panel, with mutualists in blue and yellow, cheater strain in red), while a three-member mutualistic consortium (bottom; each of the three colors indicating a different mutualistic strain) preserves all its members as the bacterial colony expands (adapted from Amor et al. [51]).

Many of the collective features observed in biofilms would not be achieved in the absence of cooperative interactions within microbes. *Bacillus subtilis* provides a very illustrative example of the interplay between cooperation and spatial dynamics and how these two elements can impact the fate of the population. Extracellular digestion of starch is a cooperative task for *B. subtilis* that requires a minimum quorum for the process to be efficient. If starch is the only carbon source available, growth in well-mixed cultures is only possible if the population size is over the minimum quorum. In contrast, in the presence of spatial structure survival is much less dependent on the initial population size, as clusters of cooperators can locally digest enough starch to sustain growth [52]; see Figure 3a. Richer population dynamics can also arise as cooperators face spatially-related conflicts. In growing biofilms, for example, cells at the periphery can starve the interior cells by consuming the available nutrients, while they can also act as a barrier that protects the interior from chemical attacks. Liu et al. [53] studied such conflict in the context of *B. subtilis* biofilms growing in limiting nitrogen conditions. The authors showed that a metabolic dependence of cells in the biofilm periphery on the ammonium generated by inner cells caused growth oscillations. The different phases driving such oscillations are as follows. Nutrient consumption during peripheral growth decreases the availability of nutrients at the interior biofilm, which is followed by a shortage in ammonium supply by the interior cells, in turn halting the growth of periphery cells and favoring the diffusion of fresh nutrients to the interior. Thus, ammonium production is restored at the interior, giving rise to a new growth cycle. Noticeably, such periodic renewal of nutrients allows the interior cells to remain alive as the biofilm grows, providing increased resilience of the population against chemical attacks. Beyond conflicts arising within clonal populations, diverse microbial ecosystems in nature typically face the challenges of a variety of social interactions among different members [7].

Synthetic biology has helped to disentangle different feedbacks between spatial structure and microbial social interactions. In a pioneer work that studied how two phenotypes compete for space in growing colonies, Hallatscheck et al. [54] showed that microbial populations can dramatically lose genetic diversity during range expansions. In a growing colony, reproduction mainly occurs at the colony edge, where there are both available nutrients and space to be occupied. Cells that are close to the edge then compete for their progeny to reach the available space, a successive founder effect making some lineages progressively excluded from the advancing edge of the colony [54,55]. Later studies showed that mutualistic interactions oppose this demixing process [26,44]. The top panel in Figure 3b shows the spatial demixing of two competing phenotypes in a yeast colony: the blurred-color at the center is the result of a well-mixed inoculum that originated the colony, and sectors containing a single phenotype (either blue or yellow) become progressively more apparent moving towards the edge of the colony. The lower panel in Figure 3b exhibits an analogous arena for a pair of mutualists. In this case, growth is enhanced at locations in which the different partners exchange the mutualistic goods efficiently, so that the two strains remain relatively mixed as the open space is colonized. The combination of the serial founder effect and mutualism not only gives rise to a characteristic width of the single-strain sectors [44], but can also lead to counter-intuitive effects such as a slower range expansion speed in nutrient-rich environments [51].

Synthetic model systems recently validated that spatial structure can help microbial cooperators thrive against cheaters, in agreement with earlier theoretical predictions [18,56,57,58,59]. Using synthetic yeast populations, Van Dyken et al. [60] showed that cheaters achieve high population fractions in well-mixed scenarios, whereas cooperators increase in frequency during range expansions; see also [61]. Analogous dynamics take place when cheaters challenge mutualistic assemblies. As shown by Momeni et al. [62], positive feedbacks between mutualists promote growth in locations where they are abundant, whereas regions experiencing a higher exploitation by cheaters endure an overall fitness decrease. Therefore, mutualists increase in abundance during range expansions, and cheaters are progressively left behind. Analogous results were observed for different synthetic bacterial models [50,51] suggesting that, beyond specific features of the model system, spatial self-organization can generically help mutualists resist exploitation by cheaters (Figure 3c).

## 4. Towards Higher Complexity in Synthetic Microbial Communities

Microbial communities [63] are formed by many different microorganisms interacting with each other in a shared environment. Their different members often compete for limited resources or even engage in chemical warfare. Other widespread processes such as cross-feeding of resources, division of labor or niche construction indicate that mutualism and commensalism also play a key role in shaping the dynamics and influencing the stability of microbial ecosystems. From an engineering perspective, how can we deal with the complexity of such interaction networks in order to manipulate microbial communities?

**Figure 4:**
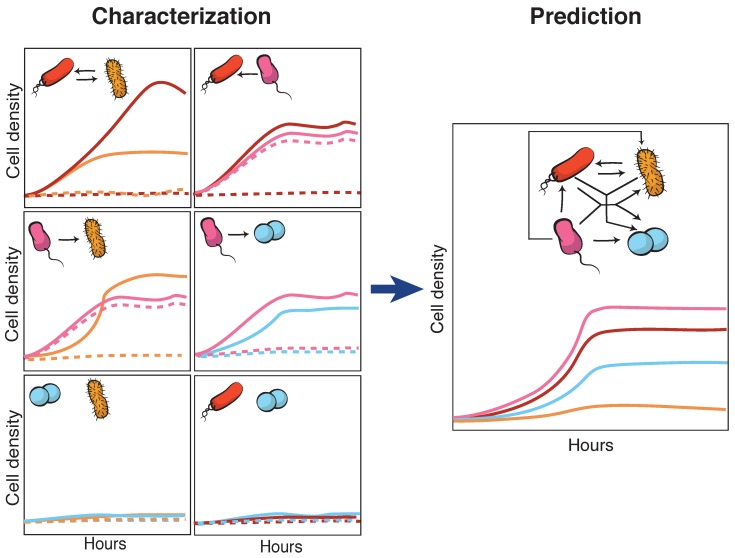
Bottom-up assembly of synthetic communities. Bottom-up approaches to understand microbial community assembly [64,65] aim at predicting the assembly of multispecies communities (right-side panel) based on the features of simpler subsets of the community (two-member cocultures on the left-side panels). In these schemes, dashed lines represent hypothetical time series for monocultures, and solid lines stand for the corresponding cocultures. The four-member mutualistic network on the right panel was inspired by one of the microbial consortia in [64].

A strategy to approach this major challenge [63] relies on tackling community complexity in a progressive manner. By first building detailed understanding of relatively simple subsets of the community, the aim is to achieve a high predictive power when combining different building blocks, i.e., multiple community subsets. Kong et al. [64] successfully adopted this approach to predict the dynamics of three- and four-member communities organized into different interacting networks. They engineered the native modular pathways of two antimicrobial molecules (namely, lysin and lactococcin A) in synthetic *Lactococcus lactis* to generate a wide range of microbial interactions including mutualism and competition. The authors were then able to obtain a very close match between their theoretical model (which gathered information of the dynamics of pairwise interactions) and the observed community dynamics for different consortia with higher members. These findings align well with previous results by Friedman et al. [65] in which the competition outcomes of two-species laboratory ecosystems were used to predict the survival of the different members in a larger community context, including eight and even 20 interacting species [66]. Although not describing the community dynamics, their approach provides intuition on the outcome of the assembly process: a given species should survive in a given consortium as long as it survives all the possible pairwise competitions with the different members. The scheme in Figure 4 displays how such bottom-up approaches use the characterization of simple communities in order to predict the behavior of more complex ones.

Non-linearities (e.g., high-order interactions [67] in which the presence of one species affects the interaction between others) in community assembly could dramatically reduce the validity of the bottom-up approach. For example, when increasing the number of cross-feeding interactions in synthetic *E. coli* consortia, the efficiency of three-member mutualisms can show significant epistasis, meaning that the three-member performance differs from linear predictions based on the efficiency of the two-member mutualisms. It is noteworthy that the number of consortia displaying epistasis remained a small minority of the cases. In a similar way, analysis of naturally co-occurrent species in laboratory environments revealed that higher order interactions in microbial community assembly are rare. While potential higher order interactions will have to be assessed in more complex microbial consortia, both natural and synthetic [68], their apparent rarity suggests that the bottom-up approach could provide useful insights into community engineering.

Beyond achieving stable synthetic assemblies, a central aim in community engineering is to design novel (or improved) community functions. Cooperative interactions are very often key components of such designs, as they can lead to emergent collective features that none of the building blocks display. For example, Kim et al. [69] constructed an artificial mutualism between *Azotobacter vinelandii*, *Bacillus licheniformis*, and *Paenibacillus curdlanolyticus* in which each species accomplished a different ecological task, namely nitrogen fixation, penicillin degradation, and digestion of cellulose. It is noteworthy that the three-member consortium was successful in an environment where none of the three species would survive alone. More recently, Hay et al. [70] designed a synthetic light-driven consortium by co-culturing the cyanobacteria *S. elongatus* with different heterotrophs. In this case, the consortium was able to generate different biochemical outputs according to the metabolism of the heterotroph included. Instead of just a one-way cooperation (the autotroph providing a carbon source to the heterotroph), mutualistic interactions prevail in these artificial photosynthetic communities as the heterotroph benefits the cyanobacteria by reducing oxidative stresses [71]. Alternatively, division of labor via distribution of the metabolic pathway among synthetic consortia has been shown to enhance productivity in laboratory ecosystems [72]. Novel ways to engineer mutualistic consortia could be guided by both theoretical [73] and experimental advances (e.g., Conjugative Assembly Genome Engineering (CAGE) [74] or CrispR/Cas9 multiplex genome engineering [75]), potentially improving our control over synthetic microbial communities.

Beyond assembling stable synthetic consortia, community engineering can also focus on driving target communities to alternative stable states using minimal interventions. In that sense, potential feedbacks between the fitness of a species and its ecological function can lead to convenient community dynamics motifs such as function-and-die [76,77], where the synthetic species would die if its action is not required. Experimental results by Mallon et al. [78] support the idea that unsuccessful invaders could be used to induce long-term effects in a community in which the invader can only remain for a short period of time. From a community engineering perspective, such unsuccessful invaders could act as drivers of community dynamics that induce a shift towards an alternative stable state [79], the invader going extinct after the shift has occurred. Beyond this, engineers could leverage horizontal gene transfer [80], using microbial invaders to introduce specific genetic traits or functions into the native community. Alternatively, transitions between mutualism and parasitism could help to control the impact of an engineered strain in a consortium. Besides the results described in Section 3, Amor et al. [51] also showed that, for a three-member synthetic consortia engaged in a range expansion, the spread of a given engineered strain can depend on whether it establishes mutualistic interactions with its partners. In particular, the whole consortium was able to spread together in harsh environments containing antibiotics (in which the three members relied on a complex mutualistic network to survive). However, one of these strains was excluded from the community as its interactions transitioned to parasitism in more favorable environments. In this way, the impact of an engineered species on the community can be constrained to conditions in which a given function is needed. Similar synthetic interaction motifs could be used to design future bioremediation or biomedical (e.g., use of probiotics) interventions.

## 5. Synthetic Mutualism in Host-Associated Communities: The Nematode *C. elegans* as a Model System

Microbial communities are often found in association with host organisms, inhabiting their alimentary tract, skin, and mucosae [81]. While the host provides an adequate environment for these microorganisms to live in, in many cases, the microbiota can benefit the host by performing different functions such as digestion of nutrients, provision of metabolites, and resistance to pathogens [82,83,84,85]. Despite the recent blooming of host–microbiome research, a number of open questions still remain in this field. How does the presence of a host organism impact the assembly of microbial communities? What are the best strategies to engineer host-associated microbiomes in order to benefit the host? In this section, we review how such questions are being addressed from a synthetic ecology perspective, with a focus on a recently-proposed model system to study the gut microbiome: the nematode *Caenorhabditis elegans*.

Most of our knowledge about the gut microbiome has been developed through a top-down approach that interrogates highly complex communities to understand host–microbe dynamics. Examples of these complex communities are those found in human microbiome samples [86] and mammalian (mostly murine) experimental models [87]. Such an approach has helped in discerning microbiomes associated with healthy individuals from those leading to disease, as well as identifying universal patterns underlying the dynamics of the microbiota associated with different individuals [88]. Moreover, interventions using antibiotics, probiotics, and fecal transplants have allowed eradicating unwanted members of the communities. Alternatively, the potential for the microbiome to accomplish novel functions has been shown via the introduction of new community members. Different engineered bacterial strains, from *E. coli* to more abundant gut commensals [89,90], have experimentally been introduced in gut communities in order to deliver therapeutic molecules [91] and, in other cases, to detect cancer [92,93,94], inflammation [95], and signals secreted by pathogens [96,97]. However, the top-down approach appears less suitable to identify the different interactions between members of the microbiome, to understand community assembly processes, or to study specific mechanisms that drive community dynamics.

A complementary, bottom-up approach that studies simpler and more tractable gut-associated communities proved to be useful in this regard. This approach usually takes advantage of small model animals, such as flies, nematodes, and zebra fish [98], which offer a higher control over community composition. This facilitates the study of specific mechanisms and interactions driving the gut microbiota. At the same time, small animals provide a convenient set up for high throughput experiments screening a large number of individuals. We here discuss the path to engineer gut-associated synthetic microbiomes in *C. elegans*.

*C. elegans* has been recognized as an experimental model for studying host–microbiome interactions only recently [99,100,101]. This nematode is one of the best-studied multicellular organisms on Earth and the first for which we have known the full genome and connectome [102,103]. Its desirable properties as a model system include selfing hermaphroditism (allowing maintenance of homozygous cultures), transparency under a microscope, a conveniently short life cycle, rapid generation time, small size, and ease of culture [104,105]. In addition, standard techniques allow obtaining large numbers of genetically-identical, synchronized (same age), gnotobiotic (microbe-free) nematodes, ultimately allowing a high level of control of both host and environment. Another advantageous characteristic of *C. elegans* is its bacterial diet. *C. elegans* feeds by ingesting whole bacterial cells and disrupting them inside its larynx, which has a special organ called grinder [106]. However, some cells can survive the ingestion process and eventually colonize the worm intestine. Manipulation of *C. elegans* bacterial diet allows evaluating microbial interactions inside its gut, complementing studies with gut isolates in vitro (see for example [107]).

Indeed, *C. elegans* proved to be particularly suited to explore ecological mechanisms driving microbial community assembly within a host. By feeding worms with two *E. coli* strains labeled with different fluorescent markers, Vega et al. [100] showed that stochasticity at early colonization stages can yield significant heterogeneity in community composition of the intestinal microbiota. In particular, early colonization by either of the two strains can lead to a bimodal distribution of single-strain-dominated microbiomes. This outcome can be reversed by high feeding rates, which instead led to a more homogeneous distribution of intestinal microbes across the worm population. Berg et al. [108] showed instead that the assembly of the *C. elegans* gut microbiome can be host dependent and deterministic. Worms fed on different microbial communities exhibited microbiota that were more similar to each other than to the microbial community they were grown on. Later, Ortiz et al. [109] examined how multispecies microbiomes assemble in the worm gut. In agreement with simple assembly rules [65], they showed that the outcome of three-species competitions within the host can often be predicted from the corresponding set of two-species interactions. Host properties such as gut pH can, in turn, dictate the outcome of bacterial pairwise interactions, indicating that the presence of the host alters the structure of multispecies communities (see also [110]). In a similar study using zebra fish, Burns et al. [111] asked whether the neutral theory could predict the assembly of gut microbiomes. The neutral theory considers that species do not actively interact, their dynamics being driven by stochastic birth and death events [112]. Burns et al. showed that the relative importance of non-neutral events increased as the host develops from larva to adult. In summary, bottom-up approaches using different model systems, including *C. elegans*, are significantly contributing to achieving a better understanding of the assembly process and uncovering the interaction networks arising in gut microbiomes.

Synthetic microbiomes in *C. elegans* have also yielded some insights into microbial functions that benefit the host. Like humans, *C. elegans* relies on gut microbes for nutrition [113], optimal development [114], and protection against pathogens [115,116]. In a recent paper, Scott et al. [101] advanced our understanding of the role of intestinal microbes in drug metabolism. Using *C. elegans* and an engineered *E. coli* strain, they showed that microbes can bolster or suppress the effects of a family of anti-cancer drugs (fluoropyrimidines) on the worm through their metabolism. Microbes can also provide metabolites that the host might not be able to produce. For example, *C. elegans* lacks the genetic machinery to produce nitric oxide (NO), an important signaling molecule in multicellular organisms, and takes advantage of the NO generated by several *Bacilli* species on which it feeds. Gusarov et al. [117] showed that NO produced by these bacteria improves *C. elegans* longevity and stress resistance. Similarly, lactic acid species can protect the worm from the toxicity of reactive oxygen species [118]. Together, these studies highlight the versatility of *C. elegans* as a model organism to uncover cooperative interactions between the microbiota and the host and its potential to become a useful system to engineer novel microbiome functions.

Future scientific efforts should be devoted to uncovering the role that the spatial structure of the gut plays in the distribution of the microbiota, as well as at mapping the complex interaction network supporting the multispecies communities inhabiting the gut. These are crucial steps on the path towards engineering cooperative interactions able to enhance the presence of those species that are most beneficial to the host. As our understanding of the host-associated microbiome structure and function increases, novel ways to engineer cooperative tasks for the microbiome members to boost host health will be arising. While our ability to manipulate host-associated microbial communities is still in its infancy, we envision that small model animals, such as *C. elegans*, will be especially helpful in our journey to a full understanding of the microbial ecology of the gut of animals and humans.

## 6. Concluding Remarks

We reviewed recent work on microbial cooperation from a synthetic biology perspective, taking advantage of both simple ecological principles and a bottom-up approach to tackle the complexity of microbial communities. We described specific mechanisms favoring cooperation in microbial ecosystems, such as privatization of public goods and spatial structure. As we dealt with more complex community structures, we discussed emergent properties that result from cooperative interactions between two or more genotypes, e.g., the ability to thrive in environments where none of the species alone would survive. Finally, we highlighted that, by taking advantage of the cooperative interactions between microbes and simple model organisms, such as *C. elegans*, we can engineer the host microbiome to perform novel tasks.

We envision that synthetic cooperation will have several applications in the near future. From a biomedical perspective, engineering native microbial symbionts in order to enhance their cooperative abilities [119] could help to develop better therapies for, e.g., obesity, diabetes, and inflammatory bowel disease. Other potential applications of synthetic cooperation lay in the field of conservation biology, if paired with careful assessment of risks and ethical evaluations. Anthropogenic climate change, globalization, and urbanization are altering environmental conditions, causing a widespread loss of biodiversity and ecosystem functions. Engineering higher collective tolerance to a changing environment, as well as recovering ecological functions that might have already been lost could provide valuable tools to cope with the environmental disturbances arising in our present time [29,120].

## Figures and Tables

**Table 1:** Social interactions in microbes. This table presents common definitions for the different pairwise interactions. In some cases, slightly different definitions can be found within the literature, e.g., making no distinction between cooperation and mutualism.

Motif	Interaction
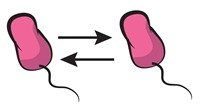	Cooperation: interaction that increases the fitness of neighboring cells. Homotypic cooperation, more specifically, refers to cooperative interactions happening between cells sharing a given genotype.
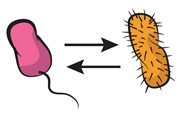	Mutualism: cooperative interaction occurring between different genotypes, i.e., heterotypic cooperation.
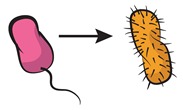	Commensalism: interaction that increases the fitness of a given genotype, with no cost or benefit for the donor.
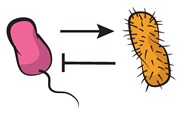	Cheating (or parasitism): one of the members benefits from the interaction at the expenses of the donor, or cooperator. This interaction motif is also known as parasitism. This same interaction motif is known as predation [34] when additional inhibitory effects, other than competition for available resources, are played against the donor organism.
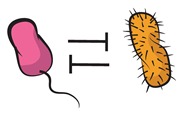	Competition: both members experience a reduced fitness as a result of the interaction.
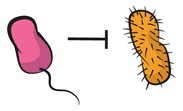	Amensalism: one of the partners is negatively affected by the presence of another, the latter experiencing neither cost nor benefit.
